# Longitudinal trajectories of Alzheimer’s ATN biomarkers in elderly persons without dementia

**DOI:** 10.1186/s13195-020-00621-6

**Published:** 2020-05-11

**Authors:** Meng-Shan Tan, Xi Ji, Jie-Qiong Li, Wei Xu, Hui-Fu Wang, Chen-Chen Tan, Qiang Dong, Chuan-Tao Zuo, Lan Tan, John Suckling, Jin-Tai Yu, Alzheimer’s Disease Neuroimaging Initiative

**Affiliations:** 1 Department of Neurology, Qingdao Municipal Hospital, Qingdao University, Qingdao, China; 2 Department of Neurology, Dalian Medical University, Dalian, China; 3 Department of Neurology and Institute of Neurology, Huashan Hospital, Shanghai Medical College, Fudan University, 12th Wulumuqi Zhong Road, Shanghai, 200040 China; 4 PET Center, Huashan Hospital, Fudan University, Shanghai, China; 5 Department of Psychiatry, University of Cambridge, Cambridge, UK; 6 Medical Research Council and Wellcome Trust Behavioural and Clinical Neuroscience Institute, University of Cambridge, Cambridge, UK; 7 Cambridgeshire and Peterborough NHS Trust, Cambridge, UK

**Keywords:** Alzheimer’s disease, ADNI, Amyloid, Biomarker, Tau, Neurodegeneration

## Abstract

**Background:**

Models of Alzheimer’s disease (AD) pathophysiology posit that amyloidosis [A] precedes and accelerates tau pathology [T] that leads to neurodegeneration [N]. Besides this A-T-N sequence, other biomarker sequences are possible. This current work investigates and compares the longitudinal trajectories of Alzheimer’s ATN biomarker profiles in non-demented elderly adults from the Alzheimer’s Disease Neuroimaging Initiative (ADNI) cohort.

**Methods:**

Based on the ATN classification system, 262 individuals were identified before dementia diagnosis and accompanied by baseline and follow-up data of ATN biomarkers (CSF Aβ42, p-tau, and FDG-PET). We recorded the conversion processes in ATN biomarkers during follow-up, then analyzed the possible longitudinal trajectories and estimated the conversion rate and temporal evolution of biomarker changes. To evaluate how biomarkers changed over time, we used linear mixed-effects models.

**Results:**

During a 6–120-month follow-up period, there were four patterns of longitudinal changes in Alzheimer’s ATN biomarker profiles, from all negative to positive through the course of the disease. The most common pattern is that A pathology biomarker first emerges. As well as the classical A-T-N sequence, other “A-first,” “T-first,” and “N-first” biomarker pathways were found. The N-A-T sequence had the fastest rate of pathological progression (mean 65.00 months), followed by A-T-N (mean 67.07 months), T-A-N (mean 68.85 months), and A-N-T sequences (mean 98.14 months).

**Conclusions:**

Our current work presents a comprehensive analysis of longitudinal trajectories of Alzheimer’s ATN biomarkers in non-demented elderly adults. Stratifying disease into subtypes depending on the temporal evolution of biomarkers will benefit the early recognition and treatment.

## Background

Alzheimer’s disease (AD) has a long preclinical phase that is characterized by accumulating pathology in the brain [1]. This pathology leads to detectable cognitive deficits in the preclinical stages of the disease [2]. The length of the preclinical phase has encouraged efforts to identify in vivo biomarkers to aid disease diagnosis and prognosis [3]. During this period, biomarkers derived from biofluids and brain imaging vary in continuous pathological processes that begin before the onset of symptoms [1, 4, 5].

In 2018, the National Institute on Aging and Alzheimer’s Association (NIA-AA) created a research framework to biologically define AD by ATN biomarkers (Aβ deposition [A], pathologic tau [T], and neurodegeneration [N]) and treat cognitive impairment as a symptom/sign of the disease [6]. Models of AD pathophysiology theorize a temporal sequence in which amyloidosis [A] initiates a biological cascade, followed by pathologic tau aggregation [T] that leads to neurodegeneration [N] [7, 8]. Besides this classic A-T-N sequence, other biomarker sequences are possible [6]. For example, primary tauopathies can evolve towards Aβ plaques before neurological signs are seen, that is, a T-A-N sequence. A current challenge in AD research is to identify the sequence of pathologic changes that occurs during the preclinical stages of disease by in vivo longitudinal study [9].

Based on this ATN classification system, our current work is investigating and comparing the longitudinal trajectories of Alzheimer’s ATN biomarker profiles in non-demented elderly adults from the Alzheimer’s Disease Neuroimaging Initiative (ADNI) using longitudinal follow-up data. The objective was to visualize which pathology biomarkers first emerge and how they change through the course of the disease. Understanding these longitudinal changes will provide insight into the pathophysiological progression of AD and potentially stratify the disease into subtypes depending on the temporal evolution of biomarkers.

## Methods

### Study design

The ADNI was launched in 2003 as a public-private partnership, led by principal investigator Michael W. Weiner, MD, VA Medical Center and University of California-San Francisco (http://www.loni.ucla.edu/ADNI). The ADNI was established to test whether serial MRI, PET, other biological markers, and clinical and neuropsychological assessment can be combined to measure the progression of mild cognitive impairment (MCI) and early AD. For more information, see http://www.adni-info.org.

### Participants

All participants included in this study were enrolled in the ADNI database (http://adni.loni.usc.edu/), a multicenter publicly funded longitudinal study of individuals with AD, MCI, and normal cognition (NC). ADNI participants were followed longitudinally, with visits every 6 months for the first 2 years, followed by annual visits. At each follow-up visit, any change to a participant’s clinical diagnosis or biomarker data was recorded in the ADNI database. Here, we restricted the present analysis to MCI and NC subjects whose biomarker data of CSF Aβ_42_ (labeled “A” biomarker), CSF p-tau (labeled “T” biomarker), and FDG-PET (labeled “N” biomarker) were all available.

**Fig. 1:**
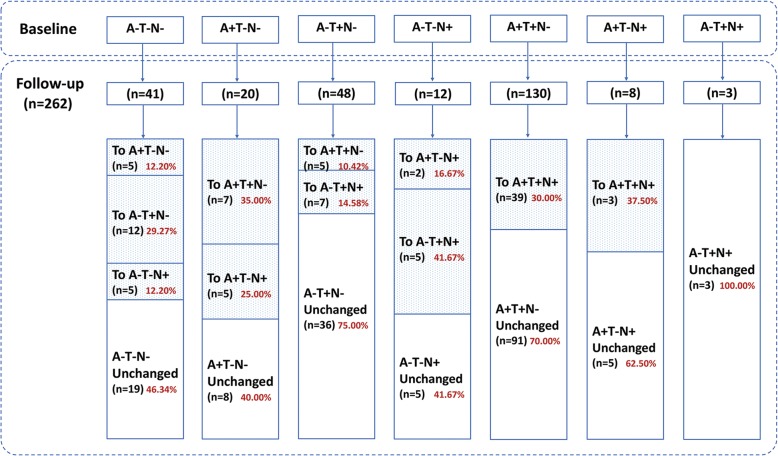
Study demographics and ATN biomarker profiles at baseline and follow-up. The analyses included 262 non-demented elderly individuals, with baseline and follow-up data of CSF Aβ42, p-tau, and FDG-PET metabolism, with seven different ATN biomarker profiles based on ATN classification. During the follow-up period of 6 to 120 months, the detailed process of the changes in ATN biomarker profiles in these individuals from the ADNI cohort was shown

In total, 262 non-demented elderly individuals, including 159 MCI and 103 NC, had baseline and follow-up data of ATN biomarkers identified from the ADNI cohort (Table 1 and Fig. 1).

**Table 1:** Demographic characteristics of study subjects

Characteristics	NC	MCI	Total
Number	103	159	262
Age (mean years ± SD)	74.98 ± 5.92	71.75 ± 7.46	73.02 ± 7.07
Gender (male/female)	59/44	91/68	150/112
Education (mean years ± SD)	16.41 ± 2.81	16.13 ± 2.54	16.24 ± 2.65
*APOE ε4* (carrier/non-carrier)	29/74	78/81	107/155

*APOE* apolipoprotein E, *MCI* mild cognitive impairment, *NC* normal cognition

### Data on CSF Aβ, p-tau, and FDG-PET

High-quality data on CSF Aβ_42_, p-tau, and FDG-PET were downloaded from the ADNI dataset. Levels of Aβ_42_ and p-tau were measured from all available CSF samples as previously described [10]. Briefly, Aβ_42_ and p-tau were measured using the multiplex xMAP Luminex platform (Luminex Corporation, Austin, TX) with Innogenetics (INNO-BIA AlzBio3; Ghent, Belgium) immunoassay kit-based research-use only reagents containing 4D7A3 monoclonal antibody for Aβ_42_, and AT270 monoclonal antibody for p-tau. All CSF biomarker assays were performed in duplicate and averaged.

The neuroimaging data of cerebral metabolic rate for glucose (CMRgl) on FDG-PET was also downloaded from the ADNI dataset. A detailed description of FDG-PET image acquisition and processing can be found at http://adni.loni.usc.edu/data-samples/pet/. The mean FDG uptake was averaged over 5 pre-defined regions of interest (metaROIs) that are sensitive to AD-related changes in metabolism, including right and left angular gyri, right and left inferior temporal regions, and bilateral posterior cingulate. PET images were spatially normalized in statistical parametric mapping (SPM) to the MNI PET template. We extracted the mean counts from the 5 metaROIs for each subject’s FDG scans at each time point, computing the intensity values with SPM subroutines. Finally, we intensity-normalized each metaROI mean by dividing it by pons/vermis reference region mean. The changes of CMRgl on FDG-PET for longitudinal analysis were observed.

### ATN classification and data collection

The cutoff values for denoting normal (negative) versus abnormal (positive) A/T/N biomarker, obtained from the extant literature [10, 11], might serve as signatures for the presence of A/T/N pathology. The cutoff concentrations of CSF Aβ_42_ and p-tau were 192 pg/ml and 23 pg/ml, respectively, while the FDG cutoff value used in this study was 1.21. Applying these cutoff values to the ATN biomarkers resulted in eight possible ATN biomarker profiles at baseline: A-T-N-, A+T-N-, A-T+N-, A-T-N+, A+T+N-, A+T-N+, A-T+N+, and A+T+N+. Those with ATN all positive (A+T+N+) at baseline were excluded from the study. Follow-up data for the other seven ATN biomarker profiles were collected (Fig. 1). We recorded the process of the first conversion in one of the three ATN biomarkers from negative to positive during follow-up. We then analyzed the possible longitudinal patterns of biomarker profiles and estimated the conversion rate and temporal evolution of biomarker changes in different patterns throughout the course of the disease. It is worth noting that, for the present study, we did not include borderline cases (± 5% from the original cutoffs for ATN biomarkers) to avoid drawing conclusions based on borderline cases. Detailed information can be found in our previously published article [12].

### Statistical analysis

Demographic characteristics of our individuals are presented using means and standard deviations (SD) for continuous variables and proportions for categorical variables. To evaluate how biomarkers changed over time, we used linear mixed-effects models. The model allowed an individual’s change rate of biomarker profiles to depend on his or her pathological stage by fitting a model with an interaction between time and “ATN” profiles. Subject-specific intercepts and slopes were included in random-effects models that allow for heterogeneity among subjects accounting for repeated measures on the same subject. Age, gender, educational years, and *APOE* ε4 genotype were also included as covariates. Estimates and 95% confidence intervals (CIs) were calculated using the parametric bootstrap method in the arm package with 10,000 replicates. The baseline estimated time (months) to each biomarker change was calculated by the absolute value of the difference between the values in individuals with follow-up data, but without this biomarker change, and the cutoff values for defining positive/negative individuals of this biomarker divided by the estimated rate of change. The total conversion time for all biomarker changes in different patterns was obtained by summing the conversion time of each of the three ATN biomarkers. All statistical analyses were conducted using the R statistical software (www.R-project.org).

## Results

The analyses included 262 non-demented elderly individuals (Table 1), with baseline and follow-up data of CSF Aβ_42_, p-tau, and FDG-PET metabolism, with seven different initial ATN biomarker profiles (Fig. 1). The follow-up time ranged from 6 to 120 months, and the median follow-up time was 24 months. The number of individuals who had follow-up data over 60 months was extremely small. Figure 1 shows the detailed process of the changes in ATN biomarker profiles.

**Fig. 2:**
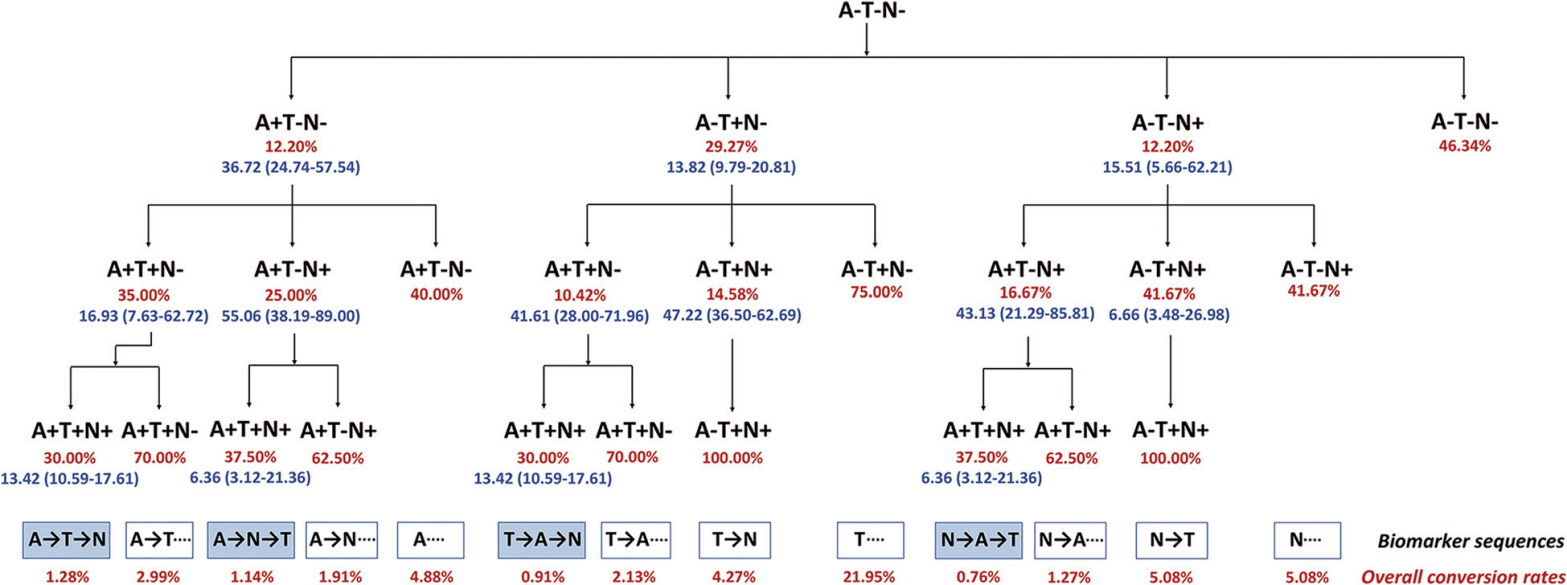
Detailed conversion rate and temporal evolution of ATN biomarker changes in different sequences through the course of the disease. The follow-up time ranged from 6 to 120 months. Red fonts represent the conversion rate at different stages. Blue fonts represent the baseline estimated time to each biomarker change. The overall conversion rates of different biomarker sequences were shown, on the basis of the current follow-up data, while 46.34% of individuals did not have any ATN biomarker changes

During the follow-up period of 6 to 120 months, there were four sequences in Alzheimer’s ATN biomarker profiles, from all negative to all positive biomarkers through disease progression (Fig. 2), specifically A-T-N, A-N-T, T-A-N, and N-A-T biomarker sequences. The overall conversion rates of these four sequences were 1.28%, 1.14%, 0.91%, and 0.76%, respectively, on the basis of the current follow-up data, while 46.34% of individuals did not have any ATN biomarker changes. The most common sequence was that the A biomarker first emerged, in line with the classic amyloid cascade hypothesis.

It is noteworthy that the T biomarker had a higher conversion rate in the first stage (29.27%), but the occurrence of the T-A-N biomarker sequence was only 0.91% in the process of total conversion. This is because the rate of subsequent conversion rate in A biomarker or N biomarker was low at 10.42% and 14.58%, respectively. Meanwhile, if the N biomarker appeared in the second stage, then there was no A biomarker change in the final stage of conversion, that is, a T-N sequence. Similarly, if the T biomarker appeared in a subsequent stage of the “N-first” biomarker pathway, then the A biomarker did not change, that is, a N-T sequence.

**Fig. 3:**
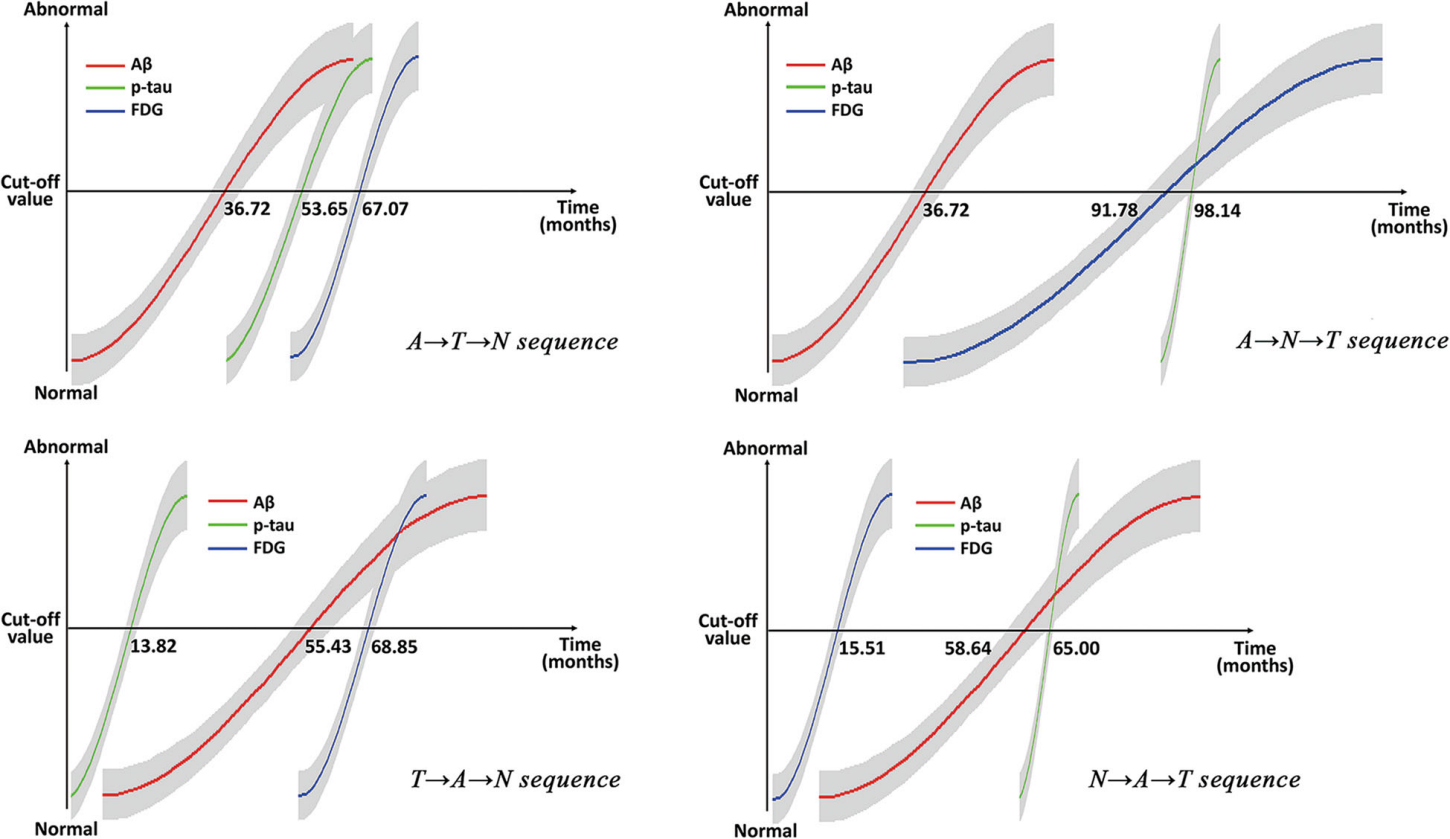
Modeling longitudinal trajectories of Alzheimer’s ATN biomarkers. The model results of the baseline estimated months to each biomarker change, reflecting the temporal evolution of pathology over the course of the disease, and the detailed time points for each biomarker change. The N-A-T sequence had the fastest rate of pathological progression (mean 65.00 months), followed by the A-T-N (mean 67.07 months), then the T-A-N sequence (mean 68.85 months), and finally the A-N-T sequence (mean 98.14 months)

The “A-first” biomarker pathway had two different evolutions: A-T-N and A-N-T. Although each is similar in the total conversion rate between these two patterns (1.28% and 1.14%, respectively), there was a significant difference in the total time for all the changes (67.07 and 98.14 months, respectively). Figure 3 depicts the model results of the baseline estimated months to each biomarker change, reflecting the temporal evolution of pathology over the course of the disease, and the detailed time points for each biomarker change. Interestingly, our results show that the N-A-T sequence had the fastest rate of pathological progression (mean 65.00 months), followed by the A-T-N sequence (mean 67.07 months), then the T-A-N sequence (mean 68.85 months), and finally, the A-N-T sequence (mean 98.14 months).

## Discussion

AD develops following a long pre-clinical phase with abnormal CSF and imaging biomarkers [1]. The ATN biomarker system is an unbiased system for grouping biomarkers and classifying research participants by the pathologic process each measures, thus provides a flexible platform to generate or test hypotheses concerning different pathologic processes and provides prognostic information of clinical change and progression [12]. Understanding the longitudinal trajectories of Alzheimer’s ATN biomarker profiles in non-demented elderly provides insight into the pathophysiological progression of AD and potentially stratifies the disease into subtypes according to the temporal evolution of the biomarkers.

The primary goal of our current analysis was to find the possible longitudinal patterns of changes in Alzheimer’s ATN biomarker profiles during the course of the disease. Our results show that there are three other longitudinal ATN biomarker pathways apart from the classical A-T-N sequence. The most common pattern is indeed that the A pathology biomarker emerges first, in line with the amyloid cascade hypothesis. The “amyloid hypothesis” has continued to gain support over the last two decades, particularly from genetic studies, and proposes that amyloid is the toxic cause of neural/synaptic damage and dementia [13]. In the current longitudinal PET study, sequential changes from Aβ to tau to cognition were clearly shown [14].

Recently, abundant human and animal data implicate both A and T in the primary pathogenesis of AD [15, 16]. The dual-pathway hypothesis suggests that A and T independently arise from pathophysiological processes that interact with pathogenic synergy [17]. The early stage of tauopathy, made up of the hyperphosphorylated tau, is a hallmark of several neurodegenerative disorders, and a “T-first” biomarker profile was found in our individuals from the ADNI cohort. The T-A-N sequence of pathological events previously proposed for late-onset AD seems to best explain the fact that the early stage of tauopathy might precede amyloid in some individuals who eventually enter the Alzheimer’s continuum when they become amyloid positive [6, 18, 19], while the T-N and N-T sequences should be considered as non-Alzheimer’s continuum profiles based on the 2018 NIA-AA Research Framework [6].

In addition, many possible etiologies and proportional mixes of etiologies (for example, cell senescence or immune dysfunction) may exist for an abnormal N biomarker finding [20]. Although N biomarkers are not specific for neurodegeneration due to AD [6], evidence has appeared to support the idea that cognitive decline and N biomarker abnormalities might precede abnormal A biomarkers in some elderly individuals who later develop AD [8, 21]. Consistent with previous work, the N-A-T sequence was also found in our individuals from the ADNI cohort. This pattern of longitudinal pathological changes needs to be further tested in living individuals by large sample prospective studies.

The second goal of our current analysis was to show the temporal evolution of biomarker changes through the course of the disease. Previous studies have found that CSF Aβ_42_ was unequivocally abnormal 5–10 years or more prior to dementia diagnosis [22], while both CSF t-tau and p-tau became progressively more abnormal as the time to diagnosis of dementia decreased [23]. Our current results are generally consistent with the above views. As can be seen in the “A-first” biomarker sequences, the A-T-N sequence had a significantly faster rate of disease progression than the A-N-T sequence. It is noteworthy that the N-A-T sequence had the fastest disease progression rate. The N biomarker is an indicator of neurodegeneration or neuronal injury that can result from many causes. Recent longitudinal analyses show that, even before amyloid turns officially positive, subthreshold accumulation correlates with subtle memory deficits and cortical tau deposition [24, 25]. Therefore, we speculate that Aβ may be already accumulating in these N-positive-first, but A-negative individuals, and increased Aβ accumulation might be associated with faster disease progression.

However, despite the long pre-clinical disease window covered by the current study, the follow-up data especially CSF data are limited because of its invasiveness. Therefore, only small numbers of participants had longitudinal data, and the majority of longitudinal participants had conversion results of biomarker changes only once. No individual had a full conversion of biomarkers from all negative to all positive across the follow-up period, and our results represent sample rather than individual effects. In addition, we calculated the conversion rates of each biomarker sequence similarly as previous research [26], while 46.34% of individuals did not have any ATN biomarker changes based on the current follow-up data. How many individuals will undergo further changes cannot be known, because of increasing death rates with age and the difficulty in longer follow-ups. Anyway, a greater number of individuals and time points, and longer follow-up time would increase the feasibility of modeling and provide more statistically powerful results in the future.

## Conclusions

To our knowledge, the current work presents a comprehensive analysis of longitudinal trajectories of Alzheimer’s ATN biomarker profiles in non-demented elderly individuals from the ADNI cohort. Except for the classical A-T-N sequence, our study has demonstrated that three other longitudinal patterns of changes in Alzheimer’s ATN biomarker profiles are also present, including the “A-first,” “T-first,” and “N-first” biomarker pathways. Understanding these longitudinal changes further demonstrates the diversity of the pathophysiological progression of the disease. Stratifying disease into subtypes depending on the temporal evolution of biomarkers could benefit the early recognition and treatment of diseases. Meanwhile, early targeted interventions blocking the effect of biomarkers might alter the natural history of the disease.

## Data Availability

The dataset generated and analyzed in the current study is available from the corresponding author on reasonable request.

## Abbreviations

Aβ: Amyloid-β
AD: Alzheimer’s disease
ADNI: Alzheimer’s Disease Neuroimaging Initiative
ATN: Amyloid-β, pathologic Tau, or Neurodegeneration
CMRgl: Cerebral metabolic rate for glucose
CSF: Cerebrospinal fluid
FDG: Fluorodeoxyglucose
MCI: Mild cognitive impairment
NC: Normal cognition
NIA-AA: National Institute on Aging and Alzheimer’s Association
PET: Positron emission tomography
p-tau: Phosphorylated tau
